# TiO_2 _Band Restructuring by B and P Dopants

**DOI:** 10.1371/journal.pone.0152726

**Published:** 2016-04-07

**Authors:** Lei Li, Fanling Meng, Xiaoying Hu, Liang Qiao, Chang Q Sun, Hongwei Tian, Weitao Zheng

**Affiliations:** 1 Department of Materials Science and Key Laboratory of Automobile Materials of MOE and State Key Laboratory of Superhard Materials, Jilin University, Changchun, China; 2 College of Science, Changchun University, Changchun, China; 3 School of Electrical and Electronic Engineering, Nanyang Technological University, Singapore, Singapore; Gazi University, TURKEY

## Abstract

An examination of the effect of B- and P-doping and codoping on the electronic structure of anatase TiO_2_ by performing density functional theory calculations revealed the following: (i) B- or P-doping effects are similar to atomic undercoordination effects on local bond relaxation and core electron entrapment; (ii) the locally entrapped charge adds impurity levels within the band gap that could enhance the utilization of TiO_2_ to absorb visible light and prolong the carrier lifetime; (iii) the core electron entrapment polarizes nonbonding electrons in the upper edges of the valence and conduction bands, which reduces not only the work function but also the band gap; and (iv) work function reduction enhances the reactivity of the carriers and band gap reduction promotes visible-light absorption. These observations may shed light on effective catalyst design and synthesis.

## Introduction

Because of its high chemical stability, high photocatalytic activity, and non-toxicity, titanium dioxide has been considered one of the more promising photocatalysts for photoelectrochemical water splitting, dye-sensitized solar absorption, degradation of pollutants, and fuel conversion. However, the undesirably fast rate of carrier recombination and the large band gap (3.0 eV for the rutile phase, 3.2 eV for the anatase phase) for photon absorption limits its practical applications.

In order to improve the catalytic efficiency of TiO_2_, one needs to consider the following three parameters: (i) how to narrow the band gap from 3.2 eV to match the energy of high-intensity visible light, (ii) how to increase the electroaffinity of the specimen to prolong the lifetime of the carriers, and (iii) how to decrease the work function to improve the reactivity of the carriers. The electroaffinity is the separation between the bottom of the conduction band and the vacuum level, which describes the ability of the specimen to hold electrons captured during reaction or to entrap the conduction electrons that localize and polarize nonbonding electrons [1]. In the process of photocatalytic, when the reduction and oxidation do not proceed simultaneously, there is an electron accumulation in the conduction band, thereby causing a fast recombination of e-h pairs. Therefore, improve the utilization rate of sunlight by modulating band gap and enhance electrons lifetime by reducing the work function and increasing the electroaffinity via locally pinning the polarized electrons are essential to promote photocatalytic reaction.

We have recently shown [2] that atomic undercoordination, such as a defect or a terrace edge formation, and nanocrystallization could improve the three quantities in the previous paragraph due to the bond order-length-strength correlation and nonbonding electron polarization (BOLS-NEP) mechanism [1]. The BOLS-NEP notation indicates that a loss of bond order shortens and stiffens the bonds between undercoordinated atoms [2, 3]. This event leads to the local densification and quantum entrapment of the bonding electrons to deepen the bottom edge of the energy bands, which polarizes the nonbonding electrons to shift up the upper edge of the bands. The conduction band edge depression increases the electroaffinity, which prolongs the carrier life. The polarization decreases the work function. On the other hand, atomic heterocoordination, such as what occurs with foreign-ion doping or codoping [4–10], surface sensitization by metal complexes or organic dyes [11, 12], noble metal loading [13, 14], and semiconductor coupling [15, 16], also exhibit the BOLS-NEP effect but to different extents.

Opinions about the B- and P-doping effects on the photoactivity of TiO_2_ remain controversial though studies have been frequent. Chen *et al*. [17] observed that B-doping widens the band gap and attributed this observation to the quantum size effect. Zhao *et al*. [18] explored a redshift in the absorption spectrum of B-doped TiO_2_ and explained the redshift as arising from crystal geometry modification of the electronic structures. In fact, B dopants can substitute for either O or Ti atoms, or can even sit in the interstitial sites. Density functional theory (DFT) calculations [19–21] suggest that B substitution for Ti atoms is energetically least favorable, while interstitial substitution and substitution for O are energetically comparable, so they can both occur in real situations. Patel *et al*. [21] suggested that at low concentration B preferentially occupies interstitial positions, but with increasing concentration B occupies lattice sites by replacing O.

Yang *et al*. [22] reported that P substitution for O narrows the band gap slightly by introducing impurity P 3p levels in the bandgap which could induce absorption edge redshift. Impurity level creation not only decreases the band gap but also provides centers promoting carrier recombination. Band gap reduction enhances visible light absorption but shortens carrier life by recombining carriers. The competition of these two effects determines the catalytic performance of TiO_2_. However, P replacement of Ti produces no impurity energy levels though it narrows the band gap of the anatase TiO_2_ slightly which is ineffective for photocatalytic improvement.

Recent progress indicates that codoping with B and P or with other nonmetals [8, 23–27] enhances UV-Vis light absorption when compared with monodoped TiO_2_ due to “p-n pairs” [8] or the “synergistic effect” [25]. However, the effects of doping and codoping with B and P on the band structure of TiO_2_ remain unclear. Here we systematically examine the effect of B- and P-doping and codoping on the electronic structures and optical properties of the anatase phase of TiO_2_ using DFT calculations.

## Materials and Methods

The present work was done using the spin-polarized DFT model of the CASTEP package [28]. The electron-ionic core interaction was represented using the ultra-soft pseudo-potential and the electron-electron interactions were described by the generalized gradient approximation (GGA) via the PW91 functional [29]. The structural optimizations were calculated using the plane-wave basis cutoff energy of 400 eV.

After structure optimization, we calculated the electronic structures and the optical properties of B- and P-doped and B/P-codoped TiO_2_. However, traditional DFT usually cannot accurately describe the band gap of semiconductors. We considered that an on-site correction can get a band gap match more in line with experimental results as well as provide a more economical calculation than a hybrid functional correction, so the GGA+U method was an efficient way to study the electronic and optical properties [30–32]. It was shown that when *U* = 4.25 eV is applied to the Ti 3d electrons, the band gap of pure anatase TiO_2_ is 3.20 eV, which corresponds well with experimental results. This *U* value has proven to be reasonable for dealing with impurity states in the bandgap [31] and in redox catalysis by TiO_2_ [33].

## Results and Discussion

### B- or P-Doping

**Fig 1:**
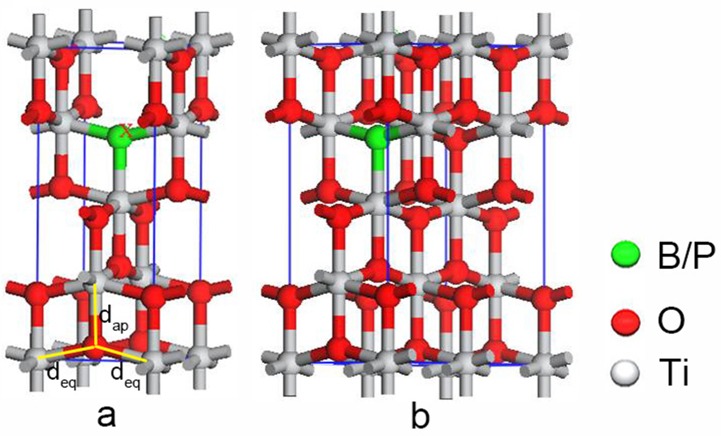
**B(P) replacement of an oxygen atom in the (a) 1 × 1 × 1 and (b) 2 × 1 × 1 anatase TiO_2_ supercells, the corresponding doping level is 8.3% and 4.2%, respectively.** The *d*_ap_ and *d*_eq_ denote the apical and the equatorial bond lengths, respectively.

We replaced one O atom with a B or P atom in the 48-atom 2 × 2 × 1, 24-atom 2 × 1 × 1, and 12-atom 1 × 1 × 1 supercells of anatase TiO_2_ shown in Fig 1 and denoted as (a) and (b) to simulate doping concentrations of 8.3% and 4.2%, respectively. Pure TiO_2_ was used as a reference. The Monkhorst-Pack mesh was set as 5 × 5 × 4, 10 × 10 × 4, and 10 × 10 × 4 for these supercells. Optimized structural parameters of *a*, *c*, and *d* (*d*_eq_ and *d*_ap_ are the equatorial and apical Ti–O bond lengths, respectively) for the primitive unit cell of anatase TiO_2_ (space group I4_1_/amd) are shown in Table 1 which fits well with experimental observations [34] and HSE06 hybrid functional calculation results [35].

**Table 1:** Optimized structural parameters of the primitive unit cell of anatase TiO_2_.

	Exp. [34]	HSE06 [35]	This work
*a*/Å	3.78	3.80	3.78
*c*/Å	9.50	9.47	9.49
*d*_eq_/Å	1.93	1.94	1.95
*d*_ap_/Å	1.98	1.98	2.00
*c*/*a*	2.51	2.49	2.51

### B-Doping

For comparison, the defect formation energy (*E*_form_) can be calculated using the following formula

$$E_{\text{form}}=E_{\text{tot}}\left(\text{doped}\right)-\left[E_{\text{tot}}\left(\text{pure}\right)+\mu_{x}-\mu_{\text{O}}\right]$$ (1)

where *μ*_*x*_ represents the chemical potential of *x* (*x* = B, P, O, and Ti) and *E*_tot_(pure) and *E*_tot_(doped) are the total energies of pure and doped TiO_2_, respectively. It is noteworthy that *E*_form_ is not fixed but that it changes with different growing conditions, such as Ti-rich, O-rich, or mixed conditions. The B chemical potential was given by the equation *μ*_B_ = 1/2*E*(B_2_O_3_)– 3/4*E*(O_2_). Under Ti-rich conditions, the Ti chemical potential is assessed from the energy of bulk Ti while the O chemical potential was obtained from the following equation:

$$\mu_{\text{Ti}}+2\mu_{\text{O}}=\mu \left({\text{TiO}}_{2}\right)$$ (2)

Under O-rich conditions, the *μ*_O_ value is taken from the chemical potential of the free O_2_ molecule, while the chemical potential of Ti is fixed by the conditions set by Eq 2. It is well known that the smaller the *E*_form_ value, the greater the stability of the doped TiO_2_ supercell. The formation energies for various concentrations of B-doped TiO_2_ are reported in Table 2. It is noted that for all concentrations of B doped into TiO_2_, substitution of the lattice O is more reasonable under the Ti-rich growth conditions and B-doping is increasingly difficult as the concentration increases.

**Table 2:** Formation energy, Mulliken populations on the B atom and the Ti(O) atoms bonded to the B atom, and bond lengths of the B–Ti(O) bonds for different B-doping concentrations.

Concentration (%)	Formation energy(eV)		Bond length (Å)			Mulliken population			Band gap (eV)
	Ti-rich	O-rich	Ti–O	B–Ti	B–O	Ti	O	B	
0	-	-	1.979/1.932(2)	-	-	1.48/1.34[22]	−0.74/−0.67[22]	-	3.2
2.1	7.09	10.69		-	1.371(2)	1.36(2)	−0.72(2)	0.27	2.84
4.2	10.89	14.49		2.352/2.074(2)		1.34(2) 1.41		−0.26	2.64
8.3	11.31	14.91		2.170/1.964(2)		1.21(2) 1.38		−0.37	0

**Fig 2:**
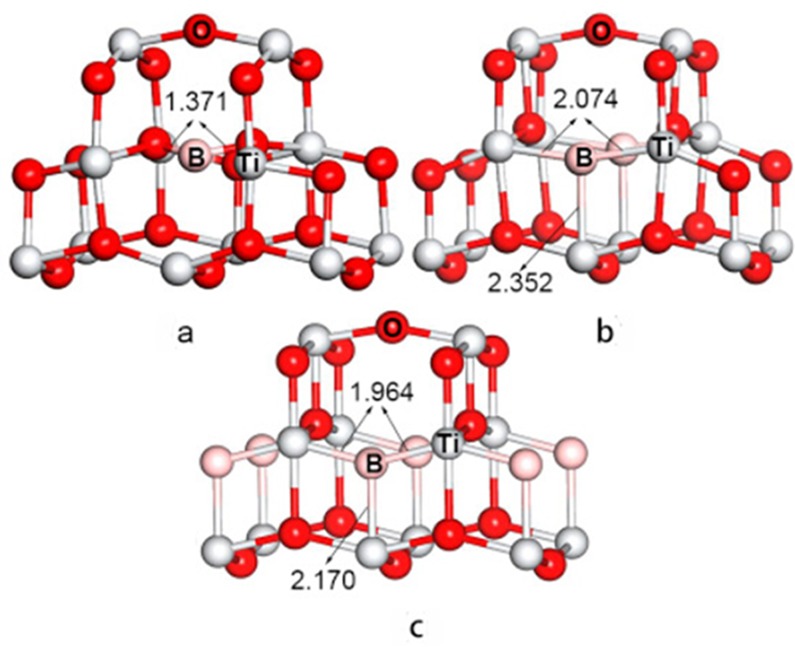
Partial geometries of the B-doped TiO_2_ at (a) 2.1%, (b) 4.2%, (c) 8.3% doping concentrations.

The partial structures derived from the optimized B-doped TiO_2_ with different doping concentrations are given in Fig 2. It is shown that high concentration (8.3% and 4.2%) doping does not distort the optimized crystal structure with elongation of the Ti–B bond. In the *d*_eq_ direction, the distances between B and Ti atoms stretch from the initial 1.932 Å to 1.964 and 2.074 Å, meanwhile, in the *d*_ap_ direction, the distances elongate from the initial 1.979 Å to 2.170 and 2.352 Å for 8.3% and 4.2% B-doping, respectively.

When the doping concentration is low (2.1%), the B atom deviates from its original position to instead bind with two neighboring O atoms to form a Ti–O–B bond in accordance with XPS measurements [21, 36]. Both B–O bonds are 1.371 Å. Furthermore, the Mulliken population study shows that the charges on the B atoms are about −0.37 and −0.26 for the 8.3% and 4.2% doping, respectively (see Table 2). The charge density is mainly distributed on the B atom rather than on the neighboring Ti and O atoms. At the 2.1% doping level, B atoms have a positive charge of 0.27 and form a Ti–O–B structure after the transfer of electrons from B to the adjacent O atoms.

**Fig 3:**
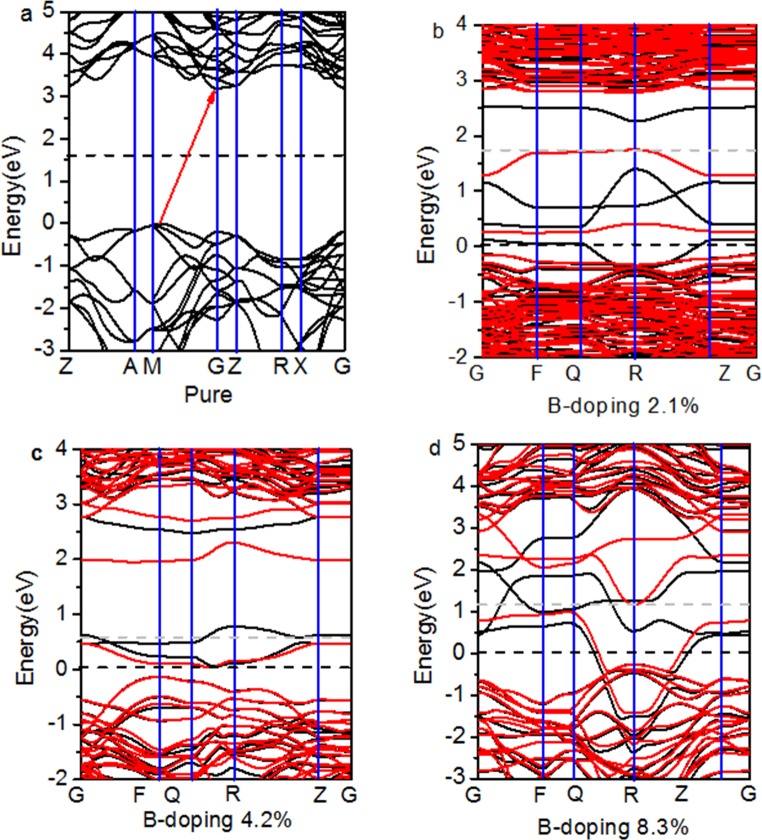
**Band structures for (a) pure and B-doped TiO**_**2**_ **at (b) 2.1%, (c) 4.2%, and (d) 8.3% doping concentrations.** Black and red lines denote the spin up and down states, respectively. The energy is measured from the top of the valence band of pure anatase TiO_2_.

The band structures of pure and B-doped anatase TiO_2_ are shown in Fig 3. The conduction band minimum (CBM) and the valance band maximum (VBM) of pure TiO_2_ appear on the gamma (G) and M points, respectively. The DFT+U calculations confirm an indirect band gap of 3.20 eV for the anatase phase. Fig 3B–3D shows that the band gap decreases gradually with an increase in the doping concentration. The band gap approaches zero at the 8.3% doping level.

**Fig 4:**
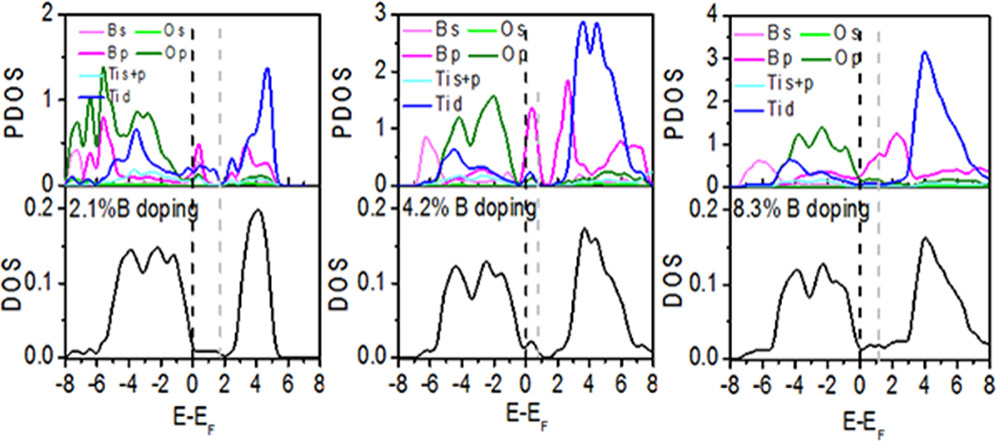
PDOS and normalized DOS for B-doped anatase TiO_2_. The energy is measured from the top of the valence band of pure anatase TiO_2_. The gray dotted line represents the actual Fermi level. PDOS is the average density of states of each atom near the defects.

Fig 4 shows the projected density of states (PDOS) near the dopants and the total density of states (DOS) for B-doped TiO_2_. The PDOS plots indicate that the O 2p states form most of the valence band while the conduction band is mainly composed of the Ti 3d states in B-doped TiO_2_, which is consistent with pure TiO_2_. B doped into TiO_2_ does not produce a simple superposition of valence electrons but the process instead exchanges and polarizes electrons. First, some impurity energy levels emerge in the bandgap and they are mainly composed of B 2p electrons and neighboring Ti 3d and O 2p electrons. Second, the strong delocalization and bonding characteristics are formed by B 2s and O 2p electrons. Third, the valence and conduction bands gradually broaden with increased concentration of B-doping.

### P-Doping

Substituting O sites with P in the TiO_2_ was also examined for the same set of anatase TiO_2_ supercells. The formation energies for P-doped anatase TiO_2_ were also calculated according to Eqs (1) and (2), in which the chemical potential of phosphorus was obtained from the equation *μ*_P_ = 1/2*E*(P_2_O_5_)– 5/4*E*(O_2_). Table 3 lists the optimal structural parameters. For all concentrations of P doped into TiO_2_, substitution of the lattice O is reasonable under the Ti-rich growth conditions and P-doping becomes increasingly difficult as the concentration increases.

**Table 3:** Formation energy, Mulliken populations on the P atom and the Ti atoms bonded to the P atom, and bond lengths of the P–Ti bonds for different doping concentrations.

Concentration (%)	Formation energy (eV)		Bond length (Å)		Mulliken population			Band gap (eV)
	Ti-rich	O-rich	Ti–O	P–Ti	Ti	O	P	
0	-	-	2.003/1.945(2)	-	1.48(1.34[22])	−0.74/(−0.67[22])	-	3.2
2.1	11.47	15.07		2.264/2.362/2.412	1.22/1.26/1.32		−0.17	3.12
4.2	12.86	16.46		2.235(2)/2.367	1.24(2)/1.36		−0.17	2.46
8.3	13.85	17.45		2.222(2)/2.318	1.09(2)/1.30		−0.26	0.75

**Fig 5:**
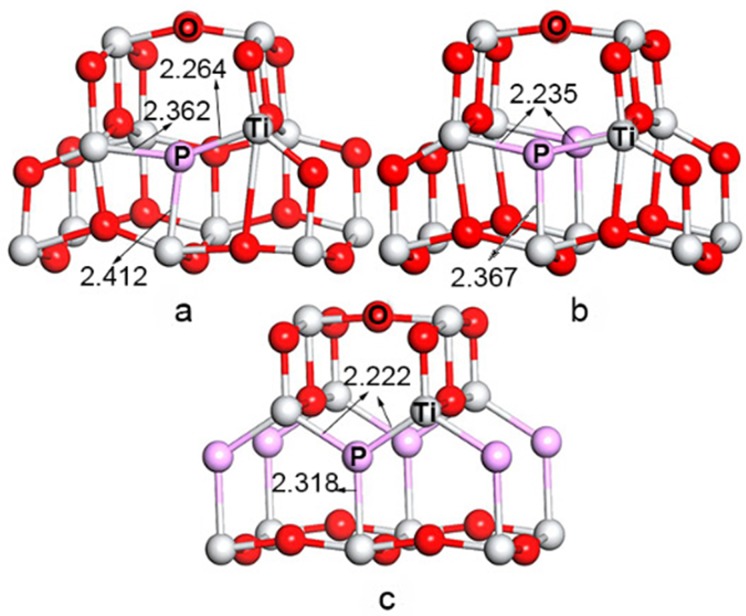
Partial geometries for P-doped TiO_2_ at (a) 2.1%, (b) 4.2%, (c) 8.3% doping level.

Fig 5 shows the local geometries taken from different P-doping concentrations in the anatase TiO_2_ supercells. This case is similar to B-doping because the distances between the P atom and adjacent Ti atoms shorten with increasing concentration, while an asymmetric structure deformation occurred for 2.1% P-doping (*d*_eq_ = 2.222 Å, *d*_ap_ = 2.318 Å for 8.3% doping level; *d*_eq_ = 2.235 Å, *d*_ap_ = 2.367 Å for 4.2% doping level; *d*_eq_ = 2.264 Å and 2.362 Å, *d*_ap_ = 2.412 Å for 2.1% doping level). These differences for both the B- and P-doped structures are larger than the original Ti–O bond length due to the larger B and P atomic radii. Moreover, considering the lower electronegativity of P than B and O, the charge on P in P-doped anatase TiO_2_ is more mobile than on B and O due to weaker ionic interactions between the P anion and adjacent Ti cation, and thus fewer electrons are transferred from Ti to P (see Table 3).

**Fig 6:**
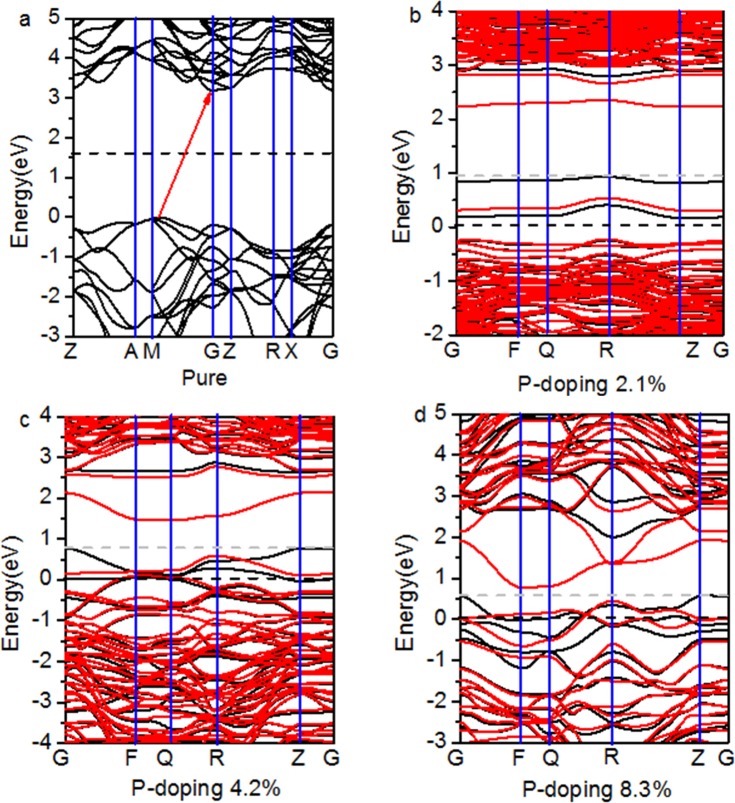
**Band structures for (a) pure and P-doped TiO**_**2**_ **at (b) 2.1%, (c) 4.2%, and (d) 8.3% doping levels.** The energy is measured from the top of the valence band of pure anatase TiO_2_.

**Fig 7:**
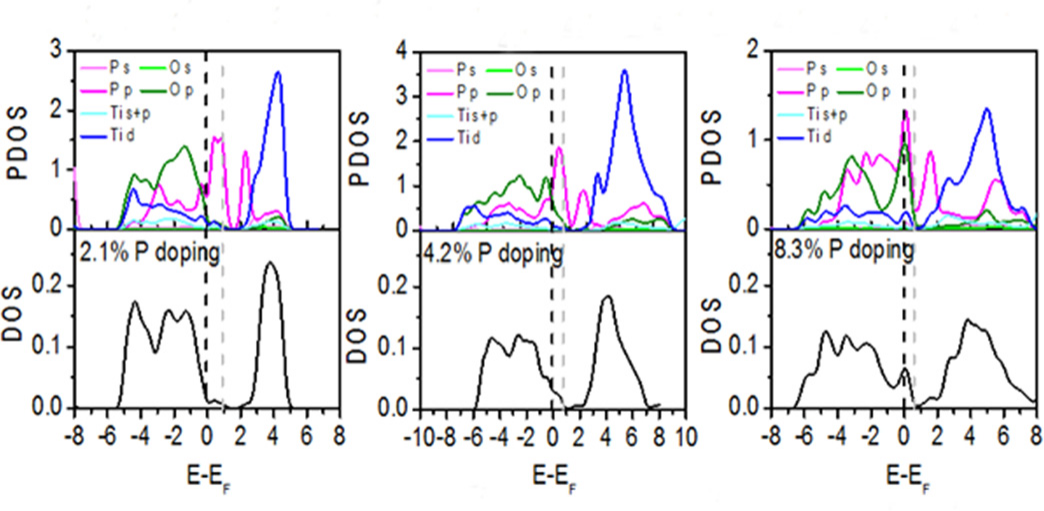
PDOS and normalized DOS for P-doped anatase TiO_2_. The energy is measured from the top of the valence band of pure anatase TiO_2_. The gray dotted line represents the actual Fermi level. PDOS is the average density of states of each atom near the defects.

Fig 6 shows that P-doping reduces the band gap to 3.12 eV for the 2.1% doping level, 2.46 eV for the 4.2% doping level, and 0.75 eV for the 8.3% doping concentrations. Fig 7 shows the PDOS and DOS due to different concentrations of dopant. Characteristics of the B-doped TiO_2_ DOS also similarly appear in the P-doped TiO_2_. P doped into TiO_2_ also exchanges and polarizes valence electrons of pure TiO_2_. First, some impurity energy levels emerge in the bandgap that are mainly composed of the P 3p electrons and neighboring Ti 3d and O 2p electrons. Second, the strong delocalization and bonding characteristics are formed by P 3p and O 2p electrons. Third, the valence and conduction bands gradually broaden with increased concentration of P-doping.

**Fig 8:**
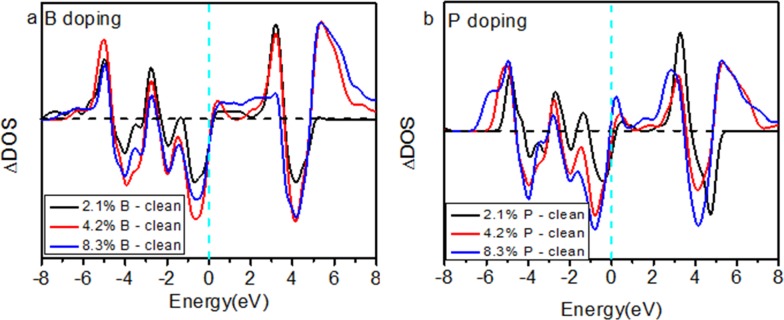
The residual DOS for B- (a) and P- (b) doped TiO_2_ at 2.1%, 4.2%, and 8.3% doping concentrations.

In Fig 8, zone-selective photoelectron spectroscopy (ZPS) technology [37] is used to determine the changes in the electronic structure for different concentrations of B and P doped into TiO_2_. The results clarified that additional DOS features are formed in all B- and P-doped TiO_2_. Entrapped states within the band gap as impurity states enhance the utilization of visible light and the antibonding states polarize the Ti 3d electrons by entrapped states, thus decreasing the work function contributing to catalytic ability, which have been proven in previous reports [38].

### B- and P-Codoping

**Fig 9:**
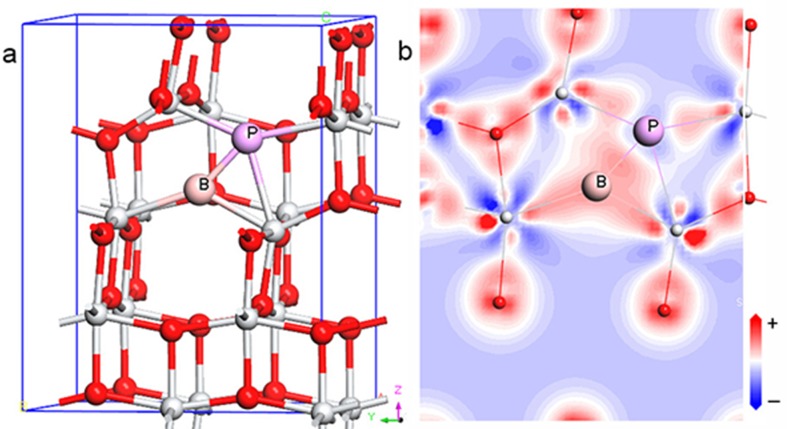
(a) Optimized geometries for the B- and P-codoped TiO_2_, and (b) corresponding difference electron density maps for the plane with B and P atoms.

To investigate the stabilities of B- and P-codoped systems, we studied the *E*_form_ for two O atoms: one replaced by a B atom and one replaced by a P atom in the 48-atom (2 × 2 × 1) supercell. Note that the increasing distance between the B and P atoms in both Ti-rich and O-rich growing conditions leads to an increase in *E*_form_. B and P atoms substituting the nearest neighboring O atoms are energetically more favorable. Fig 9 shows the optimized structure and the electron density distribution. The B and P atoms form a new B–P bond of length *d* = 1.775 Å. This bond is shorter than the sum of the atomic radii: 0.95 (B) + 1.30 (P) = 2.25 Å, which means a stronger B–P single bond is formed as compared to the original O–O separation in pure TiO_2_ of 2.474 Å. The difference charge map also shows that B gains electron from P.

**Fig 10:**
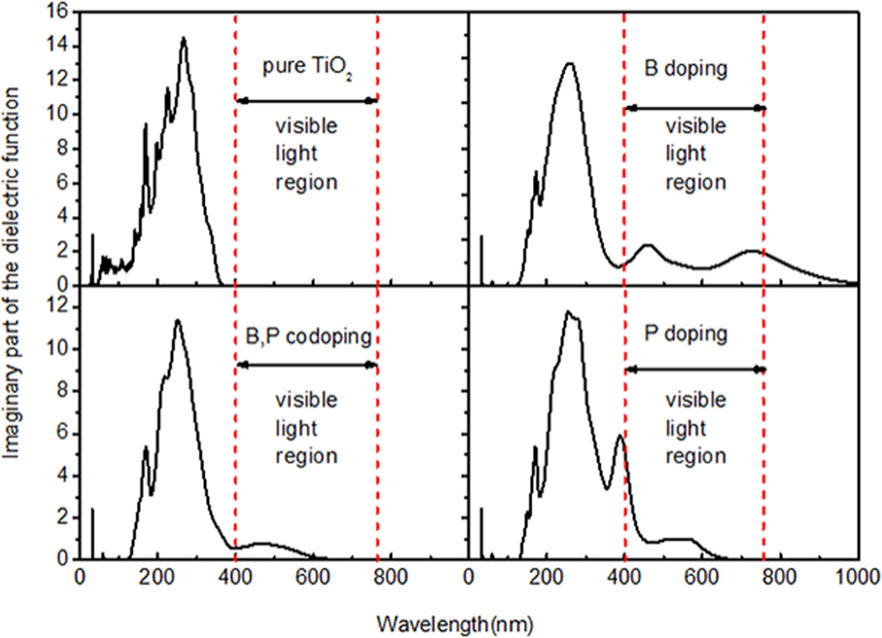
Calculated imaginary part of the dielectric function (*ε*_2_) for pure, monodoped, and codoped TiO_2_. The region between the horizontal dashed lines marks the visible light region.

To further validate the influence of doping on light absorption, we compared the imaginary part of the dielectric function (*ε*_2_), which is an important parameter for photoresponse testing (Fig 10). The calculated *ε*_2_ for pure TiO_2_ is in agreement with a recent experiment [39]. It is obvious that all B-, P-, and B/P-codoped TiO_2_ can achieve absorption of visible light and the absorption edges decrease relatively from pure TiO_2_ by 1.89, 1.32, and 1.15 eV, respectively.

**Fig 11:**
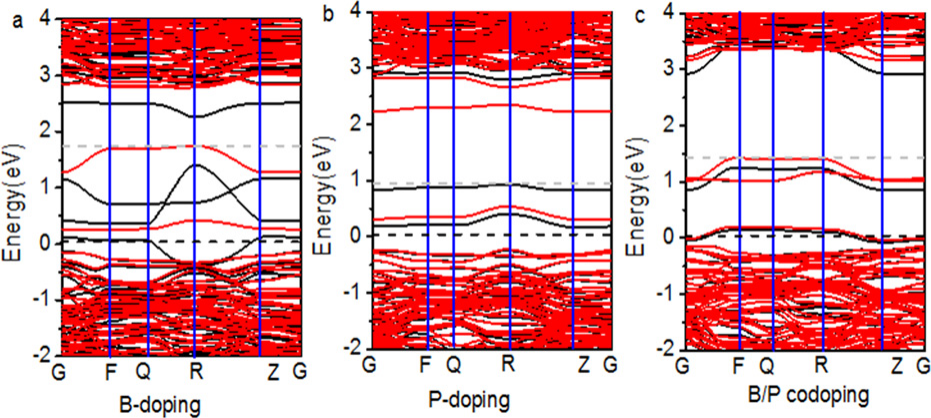
**Band structures of (a) B-, (b) P-, and (c) B/P-codoped.** The energy is measured from the top of the valence band of pure anatase TiO_2_. The gray dotted line represents the actual Fermi level.

The band structures of B-doped, P-doped, and B/P-codoped are compared in Fig 11. Because of the synergistic effect of B and P, unoccupied energy levels at the bottom of the CBM are quenched which act as a recombination center in B-doped and P-doped TiO_2_. Moreover, it is worth noting that the CBM and the VBM determine the reducing and oxidizing ability. As the VBM gets lower compared with the hydrogen production level, the oxidizing ability increases. Similarly, as the CBM gets higher, the reducing ability increases. Since the CBM of pure TiO_2_ is slightly higher than the hydrogen production level, it is best to either keep the position of the CBM unchanged or raise it in the process of band structure modulation. Because B and P passivated codoping can decrease the band gap, as well as avoid changes to the CBM compared with the B-doped and P-doped TiO_2_, codoping produces a suitable visible-light absorption region and doesn’t handicap the reducing power of TiO_2_.

## Conclusions

In summary, we investigated the effects of B-doping, P-doping, and B/P-codoping on the electronic structures and optical properties of anatase TiO_2_ by employing DFT calculations. Nonmetal doping, such as that with B and P, does not produce a simple superposition of valence electrons, but instead the process exchanges and polarizes electrons compared to pure TiO_2_. Localized entrapped states in the band gap can absorb visible light and the polarization states induced by entrapped states decrease the work function to improve the reactivity of the carriers. Moreover, B and P passivated codoping quenches the unoccupied energy levels to prolong the carrier lifetime and cause less perturbation to the CBM, thus resulting in a suitable visible-light absorption region that does not handicap the reducing power of TiO_2_.
